# Cerebrospinal Fluid (CSF) Neuronal Biomarkers across the Spectrum of HIV Infection: Hierarchy of Injury and Detection

**DOI:** 10.1371/journal.pone.0116081

**Published:** 2014-12-26

**Authors:** Julia Peterson, Magnus Gisslen, Henrik Zetterberg, Dietmar Fuchs, Barbara L. Shacklett, Lars Hagberg, Constantin T. Yiannoutsos, Serena S. Spudich, Richard W. Price

**Affiliations:** 1 Department of Neurology, University of California San Francisco, San Francisco, CA, United States of America; 2 Department of Infectious Diseases, Sahlgrenska Academy at the University of Gothenburg, Gothenburg, Sweden; 3 Institute of Neuroscience and Physiology, Department of Psychiatry and Neurochemistry, the Sahlgrenska Academy at the University of Gothenburg, Gothenburg, Sweden, Institute of Neurology, Queen Square, London, United Kingdom; 4 Division of Biological Chemistry, Innsbruck Medical University, Innsbruck, Austria; 5 Department of Medical Microbiology and Immunology, School of Medicine, University of California Davis, Davis, CA, United States of America; 6 Department of Biostatistics, Indiana University, R.M. Fairbanks School of Public Health, Indianapolis, IN, United States of America; 7 Department of Neurology, Yale University School of Medicine, New Haven, CT, United States of America; University of Nebraska Medical Center, United States of America

## Abstract

The character of central nervous system (CNS) HIV infection and its effects on neuronal integrity vary with evolving systemic infection. Using a cross-sectional design and archived samples, we compared concentrations of cerebrospinal fluid (CSF) neuronal biomarkers in 143 samples from 8 HIV-infected subject groups representing a spectrum of untreated systemic HIV progression and viral suppression: primary infection; four groups of chronic HIV infection neuroasymptomatic (NA) subjects defined by blood CD4+ T cells of >350, 200–349, 50–199, and <50 cells/µL; HAD; treatment-induced viral suppression; and ‘elite’ controllers. Samples from 20 HIV-uninfected controls were also examined. The neuronal biomarkers included neurofilament light chain protein (NFL), total and phosphorylated tau (t-tau, p-tau), soluble amyloid precursor proteins alpha and beta (sAPPα, sAPPβ) and amyloid beta (Aβ) fragments 1–42, 1–40 and 1–38. Comparison of the biomarker changes showed a hierarchy of sensitivity in detection and suggested evolving mechanisms with progressive injury. NFL was the most sensitive neuronal biomarker. Its CSF concentration exceeded age-adjusted norms in all HAD patients, 75% of NA CD4<50, 40% of NA CD4 50–199, and 42% of primary infection, indicating common neuronal injury with untreated systemic HIV disease progression as well as transiently during early infection. By contrast, only 75% of HAD subjects had abnormal CSF t-tau levels, and there were no significant differences in t-tau levels among the remaining groups. sAPPα and β were also abnormal (decreased) in HAD, showed less marked change than NFL with CD4 decline in the absence of HAD, and were not decreased in PHI. The CSF Aβ peptides and p-tau concentrations did not differ among the groups, distinguishing the HIV CNS injury profile from Alzheimer's disease. These CSF biomarkers can serve as useful tools in selected research and clinical settings for patient classification, pathogenetic analysis, diagnosis and management.

## Introduction

Infection of the central nervous system (CNS) develops early during systemic human immunodeficiency virus type 1 (HIV) infection [1]–[4] and continues throughout its untreated course [5]–[8]. The character of CNS infection, and importantly its impact on CNS function, can change over this chronic course [9], [10]. Most dramatically, in some untreated patients CNS infection evolves into an encephalitic form that presents clinically as HIV-associated dementia (HAD) with high morbidity and mortality [11], [12]. In the absence of antiretroviral treatment, chronic HIV infection is most often neurologically asymptomatic despite detectable HIV RNA and frequent inflammatory response in the cerebrospinal fluid (CSF) [7], [8]. However, mild or subclinical dysfunction in such patients may be clinically underappreciated [13], [14]. The detailed characteristics and pathogenesis of the shift from ‘benign’ meningitis to devastating encephalitis remain poorly defined, though changes in both the viral pathogen and host immune response likely contribute in concert [9], [15]. While antiretroviral therapy (ART) can prevent HAD [16]–[18] and even substantially reverse its associated neurological dysfunction [19]–[22], more subtle neurological impairment continues to be reported in virally suppressed populations with a higher prevalence in those who have suffered lower blood CD4+ T cell nadirs [14], [23], [24]. Treated patients may also show residual low-level elevations of CSF inflammatory biomarkers and viral RNA [25], [26].

Much of the understanding of the effects of HIV on the CNS derives from clinical descriptions and staging [27]–[29], patient performance on neuropsychological tests [13], [14], structural and functional neuroimaging [30]–[36] and pathological studies [12], [37]–[41]. The current research classification of these effects relies largely on neuropsychological test performance [13]. Although useful in adding a quantitative dimension and greater sensitivity to purely clinical designations, classification on the basis of neuropsychological test performance, like that using only clinical findings, has some shortcomings. These include the lack of diagnostic and pathogenetic specificity, since individuals with other disorders [14], [42] and even a background percentage of normal individuals [43], [44] may meet the impairment criteria, particularly criteria for asymptomatic neurocognitive impairment (ANI) and minor neurocognitive disorder (MND) [13]. More importantly, these criteria alone do not distinguish *active*, ongoing brain injury from *static* impairment related to earlier, but now inactive pathologies [10], [14], [45] without a clear history of progression or longitudinal assessment that may be difficult in both clinical trial and practice settings.

A complementary approach to characterizing CNS infection and resultant active brain injury in HIV infection uses CSF biomarkers for more accurate clinical diagnosis, assessment of treatment effects and understanding of evolving pathobiology in different stages of HIV-infection, and can be attained at a single sampling rather than relying on longitudinal observation [46]–[49]. Several reports document that CSF biomarkers of neuronal injury can detect active injury in HIV-infected individuals, particularly those with HAD and CNS opportunistic infections [46]–[48], [50], but also in asymptomatic patients where their detection indicates likely HIV-related subclinical neuropathology and can predict subsequent clinical presentation of HAD [51], [52].

In this cross-sectional study we compared changes in a panel of neuronal biomarkers with respect to their capacity to detect and characterize neuronal injury across the spectrum of untreated systemic disease progression from early or ‘primary’ HIV infection (PHI), through systemic progression with falling blood CD4+ T cells, to the presentation with overt HAD. We also assessed these neuronal biomarkers in antiretroviral-treated patients with plasma viral suppression and in a small group of individuals with endogenous HIV control, so-called *elite controllers* [53]–[55]. The neuronal biomarker panel included: neurofilament light chain protein (NFL), total and phosphorylated tau proteins (t-tau and p-tau), and amyloid metabolites including soluble amyloid precursor proteins alpha and beta (sAPPα and sAPPβ) and amyloid beta fragments (Aβ) ending at the 42^nd^, 40^th^ and 38^th^ amino acid of the protein (Aβ42, Aβ40 and Aβ38).

NFL, the light subunit of the neurofilament protein, is a major structural component of myelinated axons and its CSF measurement has proved to be a sensitive indictor of CNS axonal injury in a variety of neurodegenerative diseases [56]–[59] and has been previously reported to be sensitive to CNS injury associated with HIV infection [52], [60]. Tau is a microtubule-associated protein that promotes microtubule stability and is expressed primarily in non-myelinated cortical axons. The detection of increased levels of total tau (t-tau) in the CSF has been shown to be valuable in the identification of neurodegenerative disorders, particularly Alzheimer's disease (AD) [61]. When the tau protein is hyperphosphorylated, it results in the detachment of tau from the microtubules, which destabilizes the axons and can lead to self-assembly of neurofibrillary tangles [62]. This pathological process characterizes a group of neurodegenerative disorders referred to as tauopathies, which includes AD [63]. Elevations of t-tau and phosphorylated tau (p-tau) have also been reported in HIV [46], [47], [64] and other infections [48].

Amyloid precursor protein (APP) is ubiquitously expressed in neurons and undergoes sequential proteolytic cleavage by secretases, resulting in metabolites that have been linked to a variety of neuropathological consequences [65]. The cleavage of APP by α-secretase and β-secretase results in production of soluble sAPPα and sAPPβ, respectively, which are both shed from the cell membrane and diffuse into the CSF. In addition to these two APP products, we measured 3 different Aβ-amyloid peptides, Aβ42 (assessed by two different assays), Aβ40, and Aβ38. Of these, Aβ42 is the most aggregation-prone. Its lumbar CSF concentration correlates inversely with Alzheimer-associated senile plaque pathology in the brain [66], but its role in HIV-related neurodegeneration is less clear [67]–[69]. sAPPβ, Aβ38, Aβ40 and Aβ42 are soluble markers of amyloidogenic APP-processing (the pathway in which APP undergoes sequential cleavage by β- and then γ-secretase in the neuronal membrane), whereas sAPPα represents the non-amyloidogenic APP-processing in which APP is cleaved by α-secretase in the middle of the Aβ domain, precluding the formation of Aβ38/40/42 and releasing sAPPα into the brain interstitial fluid and the CSF. Decreased CSF sAPPα and sAPPβ have been described in HAD and HIV-associated CNS opportunistic infections [46], while reports of abnormalities of CSF Aβ fragments in HIV infection have been less consistent [46], [47], [64].

In this study we compared changes in these neuronal biomarkers in archived samples from pre-defined patient groups representing essential stages of HIV disease progression and viral suppression in order to establish a broad overview of neuronal change with evolving HIV infection.

## Results

The background clinical, laboratory and demographic data for each subject group are summarized in Table 1 while the fully data are included in S1 Table: Full set of the data included in the analysis. The number of subjects (N) was 20 for each group except PHI (N = 24), HAD (N = 12), treated-suppressed (N = 19) and elites (N = 8). Although median age did not differ significantly between groups, it was lower in the HIV uninfected controls and PHI subjects, possibly impacting the interpretation of comparisons, particularly NFL which increases with age in normal individuals [48]. The median nadir blood CD4+ T-cell count of the treatment-suppressed group was 60 (IQR 12–220) cells/µL while for other groups the nadir CD4+ counts were equal to or near the documented visit values (not shown). All HIV-negative subjects were recruited at the San Francisco site, and thus may not represent a comprehensive control for the other groups. Among the HIV-infected groups, the estimated length of infection did not vary significantly except for the PHI group. The majority of study subjects were men, reflecting the demography of HIV-infected persons cared for in the two medical centers from which the subjects were recruited as a convenience sample. The gender imbalance among the groups and, in particular, the low number of women in most of the groups did not allow clear separate analysis of gender effects which would demand an individual study targeting this issue to establish whether or not the results of these studies were equally applicable to women.

**Table 1:** Subject characteristics across nine groups.

		HIV negative	PHI	NA, CD4 >350	NA, CD4 200–349	NA, CD4 50–199	NA, CD4 <50	HAD	RX suppressed	Elites
Variable	Units									
***Subjects***	N	20	24	20	20	20	20	12	19	8
***Site***	SF:GOT	20∶0	19∶5	12∶8	12∶8	12∶8	11∶9	6∶6	11∶8	8∶0
***Gender***	M:F	20∶0	24∶0	16∶4	15∶5	17∶3	18∶2	12∶0	16∶3	5∶3
		median (25–75%)								
***Age (years)***	years	36 (29–48)	34 (28–43)	42 (36–44)	45 (35–53)	43 (38–54)	42 (38–49)	42 (38–47)	43 (38–49)	49 (42–55)
***Time since HIV diagnosis***	"		0.14 (0.10–0.19)	2 (0.7–12)	4 (0.7–14)	1 (0.7–12)	1 (0.1–13)	2 (4–8)	2 (0.0–8)	19 (14–23)
***Visit CD4***	cells/µL	762 (688–988)	559 (401–749)	491 (409–572)	240 (222–306)	122 (96–145)	20 (8–39)	55 (35–160)	548 (283–770)	869 (721–1070)
***Visit CD8***	"	534 (351–683)	1200 (708–1623)	909 (694–1142)	827 (694–1086)	760 (497–930)	495 (285–684)	650 (307–976)	757 (620–1094)	702 (401–828)
***CSF WBC***	"	1 (1–3)	6 (3–13)	7 (3–9)	6 (3–14)	4 (1–9)	0 (0–1)	6 (1–18)	1 (0–2)	2 (2–2.8)
***HIV plasma RNA***	log_10_ copies/mL		4.59 (4.19–5.51)	4.15 (3.35–4.62)	4.82 (4.22–5.27)	4.89 (4.48–5.20)	5.39 (4.45–5.84)	5.29 (4.79–5.71)	1.59 (1.59–1.59)*	1.59 (1.59–1.59)*
***HIV CSF RNA***	"		2.71 (1.77–3.40)	3.20 (2.47–3.63)	4.10 (3.41–4.61)	3.85 (3.50–4.38)	3.08 (2.30–3.76)	4.59 (2.71–5.02)	1.59 (1.59–1.59)*	1.59 (1.59–1.59)*
***Plasma:CSF difference***	"		2.07 (1.65–2.26)	0.99 (0.08–1.58)	0.64 (0.38–1.35)	0.99 (0.63–1.54)	2.13 (1.51–2.90)	0.78 (0.40–2.15)	0.0 (0.0–0.0)	0.0 (0.0–0.0)
***CSF: blood Albumin***	ratio	4.3 (3.4–6.2)	5.4 (4.5–7.4)	5.3 (3.9–6.4)	5.1 (4.1–7.4)	5.4 (3.6–7.5)	4.4 (3.7–5.4)	9.8 (5.8–12.8)	5.1 (4.5–6.4)	4.4 (3.9–6.0)
***QNPZ-4***	Z score	−0.10 (−0.55–0.16)	−0.16 (−0.74–0.04)	−0.07 (−0.63–0.40)	−0.16 (−0.67–0.59)	−0.45 (−1.80–0.11)	−1.42 (−12.58–−0.10)	−2.42 (−5.22–−1.32)	0.78 (0.04–1.11)	−1.08 (−1.52–−0.28)

*These log_10_ values are equivalent to the assigned ‘floor’ value of 39 copies/mL.

Our main analytic focus was on differences in the CSF biomarker concentrations across the subject groups, first considering all groups and then exploring selected *a priori* comparisons that relate to particular facets of HIV-related CNS injury with evolving infection and treatment. We also examined broad correlations across the entire dataset and across the subjects in the six viremic HIV groups.

### Group differences

**Figure 1:**
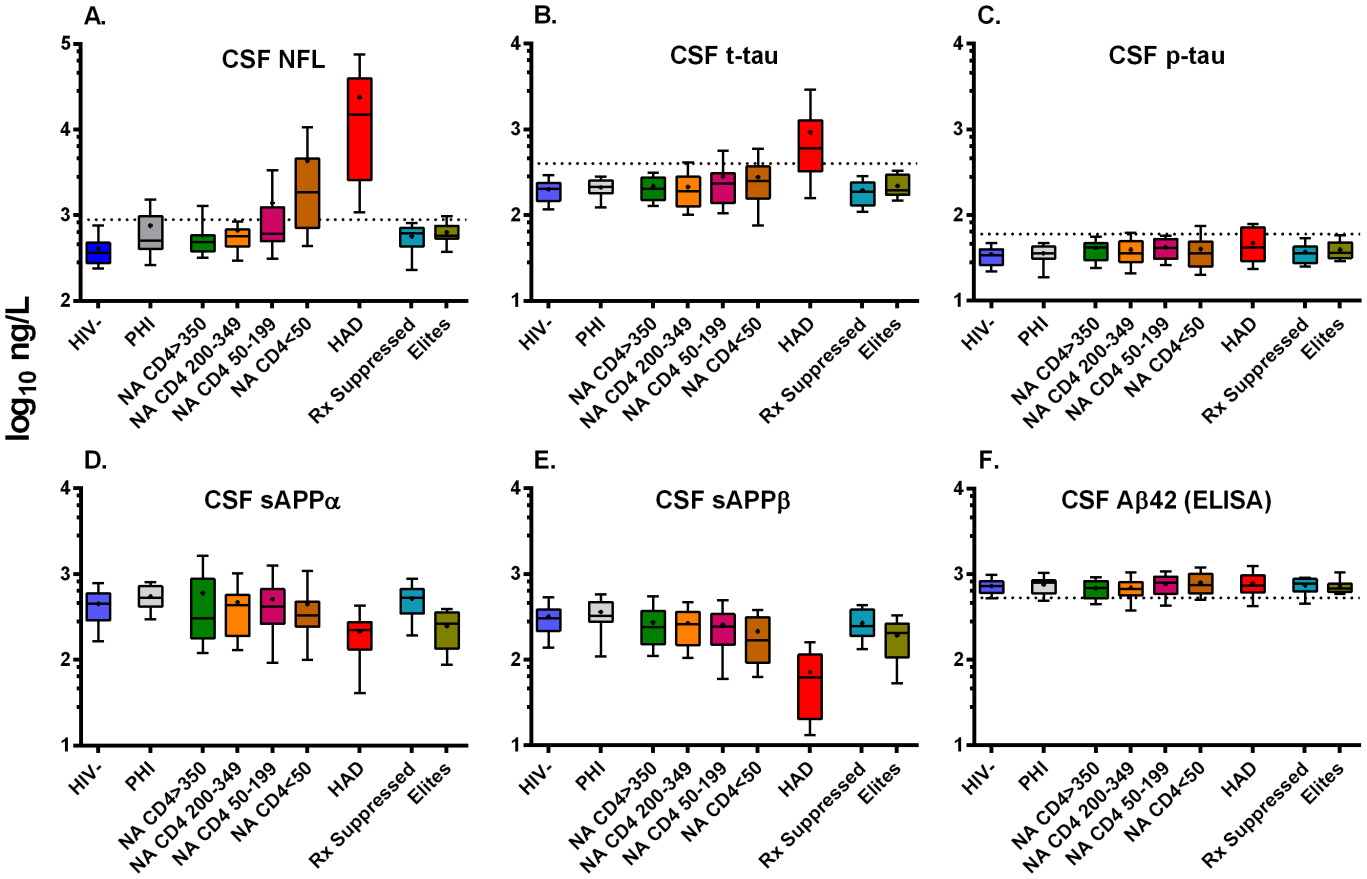
Concentrations of 6 CSF neuronal biomarkers in the 9 subject groups. Boxes in all panels depict median and IQR, whiskers show 10–90 percentiles and ‘+’ designate the means. Dotted horizontal lines show the limits of laboratory norms for the older age groups where available (upper limit of normal for NFL, t-tau and p-tau and lower limit of normal for Aβ42). Statistical comparisons of groups are given in Table 3. The biomarker concentrations are displayed in a log_10_ ng/L, over a 3 times log_10_ range, but at a higher scale range for NFL. Measured markers are listed in the title of each panel and findings described in the text. Some of the CSF NFL results included here have been previously reported in a different context [4], [10], [49], including within a larger data set characterizing CSF in relation to age [48]; here, the number of subject groups is expanded and we emphasize the comparison of NFL with the other neuronal biomarkers which have not been previously reported.

Fig. 1 shows an overview of the concentrations of the 6 main neuronal biomarkers in the 9 subject groups. Results for the Aβ-42, Aβ-38, Aβ-40 measured by the Triplex assay were similar to those of the ELISA Aβ42 (Fig. 1F) with no significant changes across the groups and are not shown.

Table 2 lists the percentage of subjects with abnormal values using age-related laboratory norms for CSF NFL, t- and p-tau and Aβ42. Because of the age differences among the subject groups, we used a regression line correction derived from a larger experience of HIV-negative controls to age-correct the comparisons of groups for NFL in this analysis [52]. Norms have not yet been defined for sAPPα, sAPPβ or the Aβ fragments 38, 40 and 42 measured by Triplex assay.

**Table 2:** Prevalence of age-related abnormal CSF biomarkers in groups.

GROUP	NFL	t-tau	p-tau	Aβ-42 (ELISA)
	% Abnormal			
***HIV-***	5%	5%	0%	10%
***PHI***	42%	4%	0%	4%
***NA, CD4 >350***	10%	10%	5%	20%
***NA, CD4 200-349***	20%	17%	15%	17%
***NA, CD4 50-199***	40%	25%	5%	15%
***NA, CD4 <50***	75%	20%	10%	20%
***HAD***	100%	75%	25%	17%
***Rx suppressed***	16%	0%	0%	10%
***Elites***	13%	0%	0%	0%

Groups and neuronal biomarkers with significant statistical comparisons using Kruskal-Wallis with Dunn's multiple comparison tests are listed in Table 3. Comparisons of p-tau and Aβ peptide concentrations (by both single ELISA and Triplex assays) as well as comparison of several of the groups were not significant and are omitted from the table.

**Table 3:** Comparisons of CSF neuronal biomarker concentrations among subject groups.

	NFL	t-tau	sAPPα	sAPPβ
	*Overall ANOVA P*			
GROUP COMPARISONS	<0.0001	0.0082	0.0011	<0.0001
	*Dunn's multiple comparisons P*			
**HAD vs. HIV-**	<0.0001	<0.01	ns	<0.0001
**HAD vs. PHI**	<0.001	<0.05	<0.01	<0.0001
**HAD vs. NA CD4>350**	<0.0001	<0.05	ns	<0.01
**HAD vs. NA CD4 200–349**	<0.001	<0.01	ns	<0.01
**HAD vs. NA CD4 50–199**	<0.05	ns	ns	<0.01
**HAD vs. NA CD4<50**	ns	ns	ns	ns
**HAD vs. RX suppressed**	<0.001	<0.01	<0.05	<0.01
**HAD vs. Elites**	ns	ns	ns	ns
**NA CD4<50 vs. HIV-**	<0.0001	ns	ns	ns
**NA CD4<50 vs. PHI**	<0.05	ns	ns	<0.05
**NA CD4<50 vs. NA CD4>350**	<0.01	ns	ns	ns
**NA CD4<50 vs. NA CD4 200–349**	<0.05	ns	ns	ns
**NA CD4<50 vs. NA CD4 50–199**	ns	ns	ns	ns
**NA CD4<50 vs. RX suppressed**	<0.05	ns	ns	ns
**NA CD4<50 vs. Elites**	ns	ns	ns	ns
**NA CD4 50–199 vs. HIV-**	<0.05	ns	ns	ns
**NA CD4 50–199 vs. PHI**	ns	ns	ns	ns
**NA CD4 50–199 vs. NA CD4>350**	ns	ns	ns	ns
**NA CD4 50–199 vs. NA CD4 200–349**	ns	ns	ns	ns
**NA CD4 50–199 vs. RX suppressed**	ns	ns	ns	ns
**NA CD4 50–199 vs. Elites**	ns	ns	ns	ns

#### NFL

The highest levels of CSF NFL were noted in the HAD group with a median value more than ten-fold higher than the HIV negative controls and the laboratory upper limit of normal (Fig. 1A). When corrected for age, all subjects in the HAD group had abnormal CSF levels of NFL, while 75% of the NA CD4 <50 and 40% of the NA CD4 50–199 also had substantial elevations. Forty-two percent of the PHI group had abnormal levels of CSF NFL, similar to the proportion noted in a previous analysis of a larger number of subjects in this PHI cohort [4]. These compared to a background of 5% elevated in the HIV- controls and lesser proportions of patients with elevations in the other HIV-infected groups with higher blood CD4+ T-cell counts (Table 2). The CSF NFL concentrations in the HAD group were significantly elevated compared to all other subgroups except the NA CD4 <50 and elites (a small and variable group) (Table 3). Interestingly, the NA CD4 <50 group were also elevated above the other groups except for the NA CD50-199 and elites. Finally, the NFL levels in the NA CD50–199 were different from the HIV negatives. These more formal comparisons support the visual impression of the significantly high level of CSF NFL in the HAD and progressive risk of axonal injury with loss of blood CD4+ T cells.

The PHI group also showed low-level CSF NFL elevation with 42 percent of subjects having concentrations above the age-adjusted laboratory norm, though in a comparison with all other groups by Kruskal-Wallis and Dunn's multiple comparison tests this did not prove statistically significant. However, an isolated Mann-Whitney comparison of these groups (asking the question formulated *a priori* whether there is an increase in CSF NFL during PHI compared to uninfected controls) showed a statistical difference (p = 0.003) consistent with previous findings in a larger group [4].

#### Tau Proteins

CSF t-tau (Fig. 1B) was elevated in the HAD group, although the magnitude of the difference from control levels and the HIV- group was less than for NFL. Using age-related reference values, 75% of HAD subjects had elevated CSF t-tau concentrations compared to lower proportions in the other groups, including 20% of the NA CD4 <50, 25% of the NA CD4 50–199 and 15% of the NA CD4 200–349. When examined statistically (Table 3) the differences between the HAD and other groups showed a pattern similar to NFL, though less robust and without a difference between HAD and the NA CD4 50–199 group. Moreover, there were no differences in t-tau concentrations among the non-HAD groups. Thus, the results show that CSF t-tau was less frequently increased than NFL and to a lesser degree. Also notable in the group comparisons was that despite elevation of CSF NFL in the PHI group, no elevation was found in t-tau in this group.

**Figure 2:**
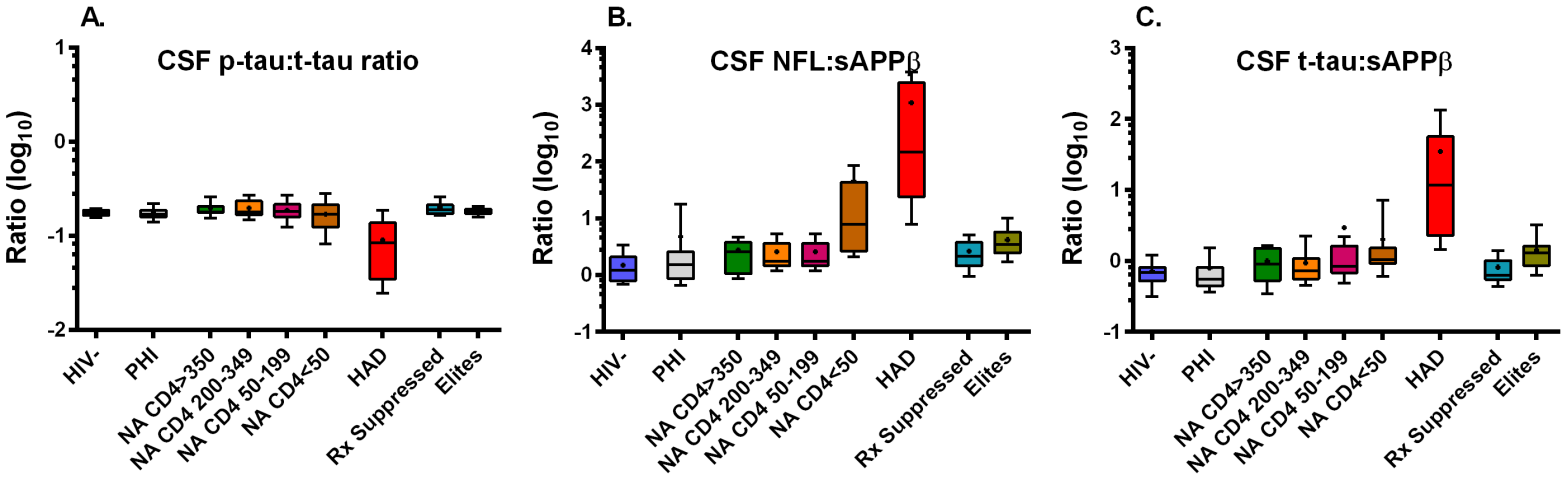
Ratios of selected neuronal biomarkers. The plots are similar in format to those of Fig. 1, but plot ratios (log_10_ differences) of selected neuronal biomarkers on log_10_ scales that differ in relation to the magnitude and ranges of the differences. A. Ratio of CSF p-tau to t-tau in the nine groups. Only the HAD group differed from any of the others in Dunn's multiple comparison test: compared to Rx suppressed and CD4 >350 (p<0.0001); to CD4 200–349 (p<0.01); and to CD4 50–199 (p<0.05). B. Ratio of CSF NFL to CSF sAPPβ. HAD group differed from HIV- and PHI (p<0.0001), from Rx suppressed, CD4+>350, CD4+200–349, CD4+50–199 (p<0.001); CD4<50 differed from HIV- (p<0.001) and from CD4+>350 (p<0.05). C. Ratio of CSF t-tau to sAPPβ. The HAD group differed from HIV-, Rx suppressed and PHI (p<0.0001), from CD4 200–349 (p<0.001), from CD4 >350 (p<0.01), from CD4 50–199 and from PHI (p<0.05); CD4<50 differed from PHI (p<0.05).

P-tau concentrations were mildly increased above the laboratory cutoff in 25% of HAD subjects but did not vary significantly across groups (p = 0.5321 by overall Kruskal-Wallis test with no significant post hoc intergroup differences). To assess the mild elevation of p-tau in HAD in relation to t-tau, we analyzed the ratio of p-tau to t-tau (Fig. 2A) which showed a tight relationship between these two tau proteins in most of the groups, but notable widening and reduction of this ratio in the HAD group (overall ANOVA p = 0.0003) with post hoc differences compared to the NA CD4 >350 (p<0.001), CD200–349 (p<0.01), CD4 50–199 (p<0.05) and treated-suppressed (p<0.001) groups. Thus, the elevation in p-tau in the HAD group was disproportionally *smaller* than the substantial elevation of t-tau, suggesting that it simply followed the latter, though the ratio was actually dampened in the HAD group indicating that there was a relative decrease in tau phosphorylation in the HAD group.

#### Amyloid Metabolites

HAD was associated with a reduction in CSF sAPPα and sAPPβ, most notably in the latter (Fig. 1 D and E) (Table 3), confirming the results of an earlier study that showed reduction of these APP cleavage products in HAD [46]. By contrast, there were no significant reductions in any of the Aβ peptides including not only the Aβ42 shown in Fig. 1F, but also using the Triplex measurements of Aβ38, Aβ40 and Aβ42 (not shown). This also confirmed the results noted in our earlier study using a different sample set [46].

Because the concentration of CSF sAPPα and sAPPβ in PHI showed a trend to elevation rather than depression as noted with advancing CD4+ cell loss, we explored the ratio of NFL and t-tau to sAPPβ as shown in Fig. 2B and C. These show that when NFL or t-tau was combined with sAPPβ as a ratio, the trend to deviation in the PHI group was reduced so that their results were nearly identical to those of the HIV negative control group. This may suggest that the neuronal injury in PHI signified by mild NFL elevation differs in character from that of the damage that occurs later in infection since it is not accompanied by a concomitant reduction in sAPPβ.

### Correlations across the entire data set

We also explored the broader correlations among the neuronal biomarkers across the entire sample set and among the six groups of untreated viremic subjects, along with their correlations with the major background biomarkers. The significant correlations of the variables among the viremic HIV-infected subjects (including PHI, four NA groups and HAD) are highlighted in Table 4 showing the Spearman's r values and emphasizing the significant correlations at two levels (r≥|0.25|and ≥|0.5|). Similar correlations were noted when all groups were included in the analysis (not shown). Thus, among the neuronal biomarkers, CSF NFL correlated positively with t-tau and negatively with the two sAPPs as anticipated by the group comparisons. Likewise, the p-tau correlated highly with t-tau. Perhaps less easily anticipated, Aβ42 correlated with t-tau and p-tau. These were positive correlations and thus quite different from Alzheimer's disease in which Aβ42 is reduced as the tau concentrations rise [66]. The reason can probably be seen in the very small increase in Aβ42 with falling CD4 counts in the NA groups and the development of HAD (Fig. 1). Finally, the two sAPPs were highly inter-correlated as previously described [70], [71].

**Table 4:** Correlations among the CSF neuronal and background biomarkers across the sample of viremic subjects.

	CSF Neuronal Biomarkers					Background Variables						
CSF Neuronal Biomarkers	t-tau	p-tau	sAPPα	sAPPβ	Aβ42	Blood CD4	Blood CD8	plasma HIV RNA	CSF HIV RNA	CSF WBC	Albumin ratio	QNPZ-4
	(Spearman's r)											
**NFL**	*0.462*	0.204	*−0.278*	*−0.349*	0.131	**−0.509**	*−0.311*	*0.415*	*0.261*	−0.186	*0.387*	*−0.315*
**t-tau**		**0.750**	−0.115	0.068	**0.566**	−0.164	−0.047	0.013	0.053	0.030	0.192	−0.141
**p-tau**			0.163	*0.369*	**0.706**	−0.012	0.076	−0.118	−0.146	−0.006	0.067	0.026
**sAPPα**				**0.791**	*0.298*	0.244	0.172	−0.085	−0.180	0.082	−0.140	*0.348*
**sAPPβ**					*0.424*	*0.381*	*0.250*	−0.248	*−0.414*	0.071	−0.180	*0.393*
**Aβ42**						−0.047	0.038	−0.145	*−0.257*	−0.119	0.039	0.048

*Italics, r>0.25 or <−0.25*; **bold r>0.5 or <−0.5.**

**Includes all viremics  =  PHI, NA, HAD.**

Of the CSF neuronal biomarkers, NFL correlated most commonly and strongly with the background biomarkers, negatively with blood CD4 and CD8 cell counts and with QNPZ-4 and positively with the plasma and CSF HIV RNA levels and albumin ratio (Table 4). sAPPβ had a similar profile of correlations though with opposite signs but not including the plasma HIV and albumin ratio.

## Discussion

This exploratory survey provides a broad view of the changes in the neuronal injury biomarkers over the course of HIV infection and treatment. The six viremic study groups were chosen to represent distinct epochs in evolving untreated systemic infection from PHI, through progressive systemic disease with CD4+ T cell loss, to the presentation with clinically overt HAD. Treatment and host endogenous (elite) viral control were also represented to assess the CNS in two types of viral control. These results extend previous findings, place them in a broader context, and both confirm the presence of neuronal injury before overt symptoms and provide a comparison of different classes of neuronal biomarkers. The changes in neuronal biomarkers across the subject groups provide a preliminary ‘reconstructed’ sequential picture of the impact of evolving infection and immunosuppression on the CNS. The aggregate findings suggest a hierarchy of injury and its detection that not only have pathogenetic implications but also potential application in the clinical evaluation and staging of HIV-related neuronal injury and response to therapy.

Among these CSF neuronal biomarkers, NFL was the most sensitive to injury and indeed demonstrated the presence of active axonal damage not only in the symptomatic HAD patients but in many of those with less apparent disease in the presence of reduced blood CD4 cells as well as during the first year of PHI. Discernable alterations in tau metabolism were largely restricted to those with HAD, while CSF amyloid proteins were also altered with reduction of sAPPβ and to a lesser degree sAPPα. The combined view of these neuronal biomarkers provides a novel characterization of the profile and evolution of neuronal injury across progressive systemic disease and viral suppression.

### Character of subject groups

#### Early infection

In some PHI patients early CNS viral invasion was accompanied by relatively mild injury reflected in the increase in median CSF NFL concentrations with 42 percent reaching abnormal levels. This increase in CSF NFL during PHI compared to HIV-uninfected subjects has been reported in more detail elsewhere [4], but the current results provide a broader context for these changes including subsequent ‘normalization’ in chronic infection with higher blood CD4+ T-cell levels (>350 and 200–350 per µL) and after treatment. Unlike the later injury of HAD, this was not accompanied by an appreciable elevation in CSF t-tau, consistent with this early injury being either milder or of a different character (for example, restricted to myelinated axonal injury rather than involving more proximal neuronal damage). Also suggesting a difference in the nature of the injury was the mild (though statistically not significant) increase in the two sAPPs in the PHI group, which contrasted with the decrease in these amyloid metabolites noted with injury during advanced infection. As a result, unlike in later disease, during PHI the ratio of NFL and t-tau to sAPPβ did not increase but remained the same as in HIV negatives suggesting that despite injury to myelinated axons there was less appreciable disruption of APP processing. Pathogenetic implications raised by these differences certainly must be considered speculative at this point and need to be tested by independent assessment involving larger samples, but they may relate to a more attenuated or different profile of inflammation compared to later infection, particularly HAD. The elevation of CSF NFL during PHI was not sustained at abnormal levels, and regressed to normal levels in those with chronic infection and higher blood CD4+ cells before again increasing with more advanced infection. Overall our comparisons suggest that PHI is associated with a phase of neuronal injury that may differ in character from chronic injury and that seems to remit, at least as reflected in this cross-sectional view. It is, of course, important to examine this issue more directly using longitudinal samples following the transition from early to chronic infection, though this is increasingly difficult to study with current treatment guidelines that recommend early ART initiation (http://aidsinfo.nih.gov/guidelines/html/1/adult-and-adolescent-arv-guidelines/0/).

#### Evolving infection with CD4 decline in the absence of overt HAD

The four chronically infected NA groups were defined by blood CD4+ T cell counts into four biologically meaningful groups in order to examine neuronal changes as systemic disease progressed. Samples were chosen from our archived experience on the basis of blood CD4+ T cell counts without reference to other clinical or laboratory variables. The higher CD4+ groups reflected earlier landmarks used to guide treatment initiation while the lower CD4+ groups related to risks of opportunistic infections and death [72]. These subjects were initially recruited as volunteers in a context that excluded patients with clinical neurological presentations or ongoing neurological evaluation. The most notable finding in the sequence of these four groups was the increase in CSF NFL with falling blood CD4 count (Fig. 1A) culminating in 75 percent of samples being abnormal in the group with <50 CD4+ T cells per µL after age correction. As anticipated from the group definitions, CSF NFL correlated with CD4 as a continuous variable among the viremic subjects. sAPPβ and to a lesser degree sAPPα also correlated with CD4 counts as noted in a previous report [51]. This indicates a substantial prevalence of underappreciated active neuronal injury in untreated patients with advanced systemic disease which likely contributes to the risk conferred by low CD4+ T-cell nadir on cognitive impairment even after treatment [24].

**Figure 3:**
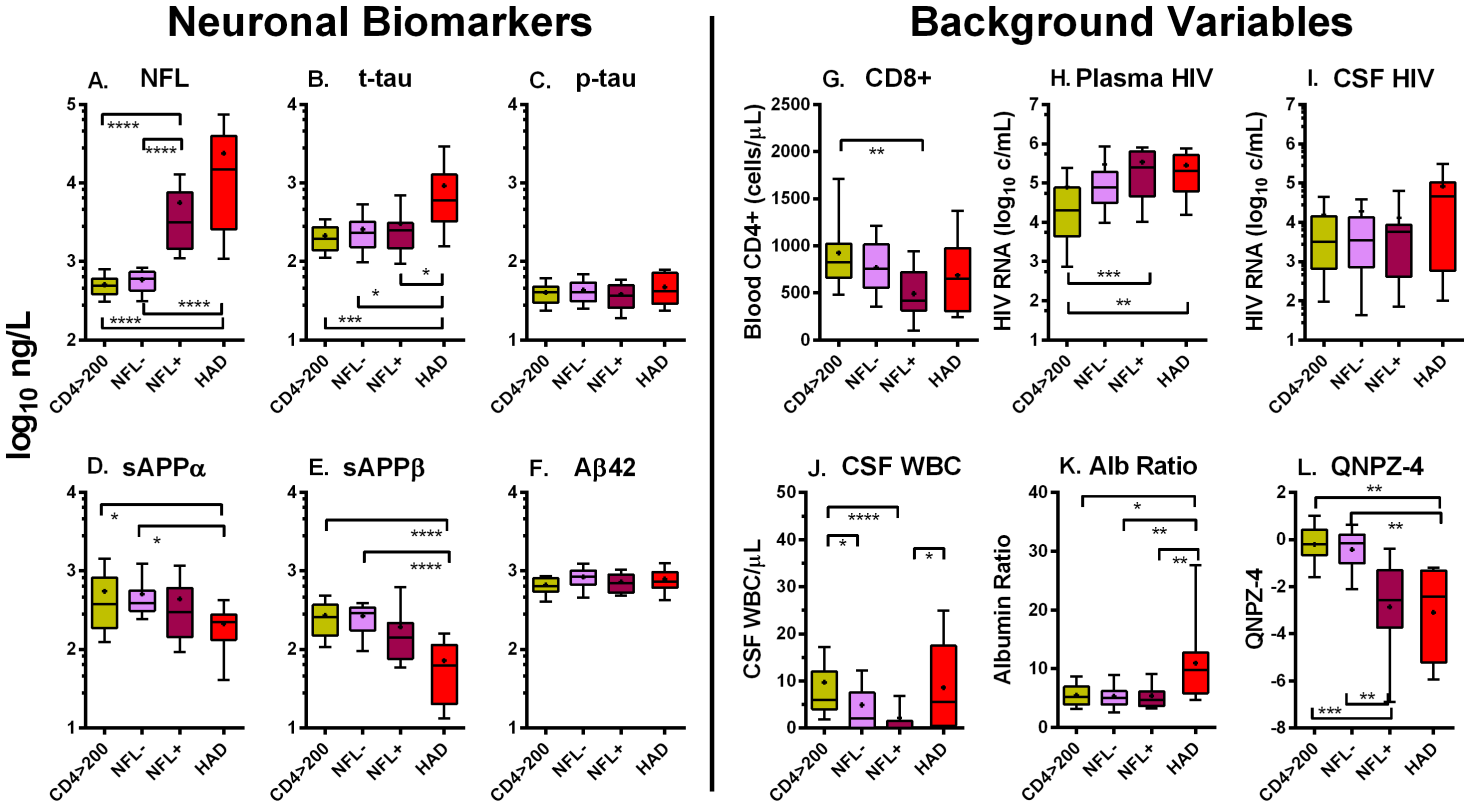
Concentrations of biomarkers in redefined NA subject and HAD groups. The left 6 panels (A–F) show concentrations of six CSF neuronal biomarkers in the four redefined subject groups discussed in text using same log_10_ axes as Fig. 1. The right six panels show some of the main background variables with vertical axes defined for each panel. The size of each re-sorted group was: CD<200, N = 40; NFL-, N = 22; NFL+, N = 18; HAD, N = 12). The horizontal brackets show p values for significant group differences by Dunn's multiple comparison test after Kruskal-Wallis test: * = <0.05, ** = <0.01, *** = <0.001 and **** = <0.0001.

In order to further examine the character of injury in NA patients, we examined CSF biomarker profiles after re-sorting the NA subjects on the basis of both CD4 and NFL into three new redefined groups: subjects with blood *CD4* >*200* cells per µL (combining the initial >350 and 200–350 groups); those from the <50 and 50–199 groups with normal CSF NFL (designated *NFL-*); and those from these same two low CD4 groups with CSF NFL above the age-related laboratory cutoff (designated *NFL*+). This importantly separated the subjects without and with detectable active axonal injury. We then compared these new groups and the HAD group for differences in the neuronal biomarkers and background variables as shown in Fig. 3. This analysis emphasizes that the NFL+ group, while similar to the HAD group in some respects, clearly differed in others.

As anticipated by the definitions used in the re-grouping, CSF NFL concentrations were high in the NFL+ group, and lower but not significantly different from the HAD subjects (Fig. 3A). Perhaps more interesting was the finding that t-tau in the NFL+ group did not differ from either the CD4 >200 or the NFL- groups, clearly distinguishing it from the HAD group (Fig. 3B). Subclinical or milder injury was thus not detected by CSF t-tau analysis. The concentration of sAPPs in the NFL+ group was intermediate, statistically differing neither from the HAD nor from the CD4+>200 or NFL- groups (Fig. 3D and E). This regrouped analysis supports a hierarchy of biomarker ‘subclinical’ HIV-related injury detection, most sensitively detected by elevated NFL, partially by sAPPβ and sAPPα, but not apparent with t-tau. Neither p-tau nor Aβ42 differed among these four groups (Fig. 3C and F).

Examination of the background variables after this regrouping also emphasized some of the salient features of the NFL+ group and its distinction from the NFL- groups, on the one hand, and HAD, on the other. The NFL+ group was distinguished by low CSF WBC counts (Fig. 3J) which were characteristically elevated both in chronic HIV infection with higher counts and in the HAD group. This may relate in part to the lower blood CD8+ (Fig. 3G) and CD4+ cells (not shown) since these cell types are the main cell constituents of CSF [73] but also to other factors governing cell traffic. Plasma HIV RNA concentrations (Fig. 3H) were similar to those in HAD and higher than in subjects with higher CD4 counts, while CSF HIV RNA did not differ across the four groups (Fig. 3). Notably, the albumin ratio in the NFL+ group was normal and did not differ from the two NFL- groups, providing another characteristic distinguishing it from the HAD group which showed frequent damage to the blood-brain barrier (Fig. 3K). Performance by the NFL+ San Francisco subjects on the QNPZ-4 testing was impaired, indicating that while this group did not present with HAD, they were clearly abnormal with a median performance similar to the HAD group. While full testing recommended for applying the Frascati criteria [13] was not done, the clear impairment of performance on this short battery likely placed these patients within the Frascati classification framework of HAD and ANI/MND. Hence, while symptoms may have been minimal or underappreciated, these individuals with elevated CSF NFL were functionally affected and might more appropriately referred to as suffering ‘quiet’ or obscured CNS injury than being ‘neuroasymptomatic’.

#### HAD

**Figure 4:**
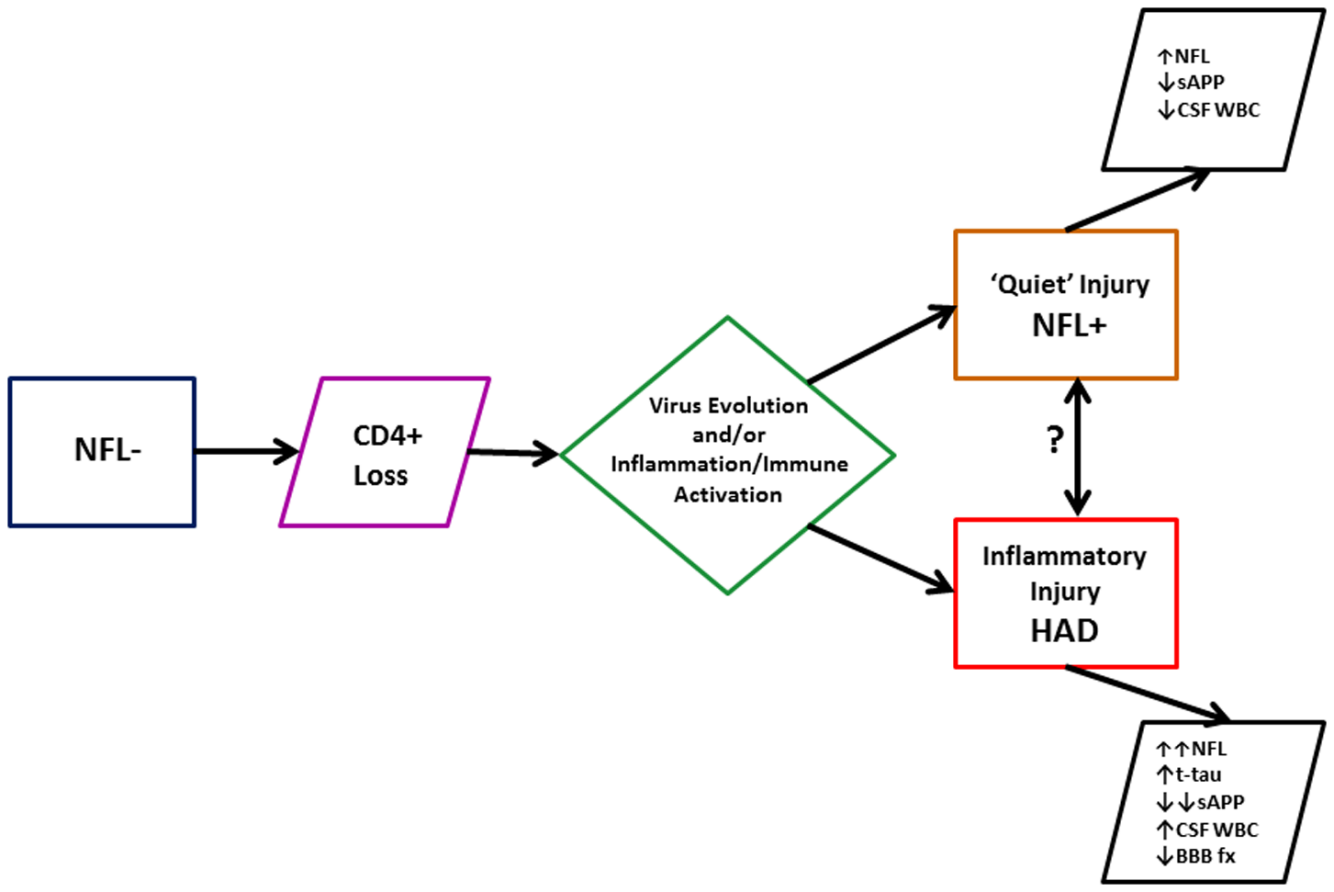
Progression to severe HIV-related CNS injury. The figure outlines a simple diagram of transition from asymptomatic NFL- infection to either ‘quiet’ NFL+ injury or HAD. It emphasizes that both have CD4+ T-cell loss in common but then diverge for undefined reasons, possibly related to viral evolution of host inflammatory/immune activation characteristics. Further study is needed to understand the factors operating in this transition. It also has not been defined whether either of these two injury states can transition to the other.

Given some of the similarities of the NFL+ and HAD groups, it might be questioned whether there was really any difference between these groups and that their clinical distinction was simply a matter of under-recognition. However, in addition to their different clinical presentations which determined the differences in diagnosis prior to the study, there were distinct biological differences that distinguished the HAD group from the NFL+ group, including significantly elevated CSF t-tau, lower sAPPα and sAPPβ, elevated CSF WBCs, and raised albumin ratios. These indicate a clear difference in both neuronal biomarker profiles and in the levels of inflammation and altered blood-brain permeability. The comparison between the NFL+ and HAD groups suggests two forms or severities of major brain injury, one with a paucity of inflammation and the other more inflammatory. In HAD the background neural injury related to infection may be augmented by more robust inflammation and breakdown of the blood-brain barrier leading to neuronal body injury or death with t-tau release and the clinical presentation of subacute neurological deterioration. A simple pathway to these two types of injury is diagrammed in Fig. 4. This emphasizes that advanced systemic disease and CD4 loss is a prerequisite for both ‘quiet injury’ (NFL+) and clinically overt HAD, but that uncertain processes favor one or the other: perhaps changes in the character of the virus or in factors that determine the profiles of inflammation. Whether either of these two forms can transition to the other is also uncertain, though detection of NFL in neuroasymptomatics has been reported to predict development of HAD [51]. These distinctions are important in understanding the links between CNS infection, consequent injury and clinical presentation, and need to be examined further.

#### Treatment suppressed

The samples for this patient group were selected on the basis of sustained blood viral suppression. Their CSF neural biomarker concentrations were normal except for 3 subjects with age-corrected concentrations just above the normative cutoff resulting in a higher median concentration than controls, though not statistically significant. Elsewhere, in an analysis of a larger sample, it has been suggested that CSF NFL in treated patients may remain above age-adjusted controls, though still within the normal laboratory limits [52]. Measurement of the other neuronal biomarkers did not detect any differences in these patients from controls, providing reassuring evidence of normalization and protection from more severe active neuronal injury in the face of treatment. While these individuals were studied only cross-sectionally, their low nadir CD4+ counts (median of 60 cells per µL) might suggest that before treatment some had sustained neural injury if they were similar to the proportion of the subjects studied here with similar blood CD4+ counts. Thus, it can be speculated that they had responded to treatment with improvement in both performance and CSF biomarkers, indicating a therapeutic effect on brain infection and consequent injury.

#### Elites

The small group of elite controllers included in this study performed relatively poorly on QNZP-4 testing with a median score of −1.1. Whether this was a consequence of HIV and its control by host mechanisms is uncertain [53]. The concentrations of the CSF neuronal biomarkers were within normal limits except for elevated CSF NFL in one of the subjects after age correction. Because the sAPP concentrations tended to be lower in this group, the ratios of NFL and t-tau to sAPPβ (Fig. 2B and C) were mildly increased and the NFL to sAPPβ ratio approached significance in comparison to the HIV- group (p = 0.053) in the Dunn's multiple comparison test. Whether this signals mild ongoing neuronal injury in this group will require additional independent study with a larger number of subjects and controls. These are an unusual group of subjects and in most the biological basis of host resistance to viremia was not known and conceivably may also carry neurological vulnerability. The ‘cost’ of protecting the brain from HIV, for example with a robust CD8+ antigen-specific CD8+ T cell response, may carry a neurological liability.

### Hierarchy of injury and detection

The differences in the CSF neuronal biomarkers in the various subject groups indicate a hierarchy in their capacity to detect CNS injury, with NFL > sAPPs > t-tau. An important question is whether these differences reflect mainly test sensitivity related to their CSF metabolism (including their release and diffusion into CSF, degradation within, or diffusion out of the CSF space) and signal properties (measurement sensitivity and accuracy), or whether they indicate a hierarchy of HIV-related injury pathways. We cannot definitely answer this question from our data, though measurements of the markers were within the quantitative range of the assays and the differences in biomarker ratios among some of the groups may favor the latter.

#### NFL

NFL was clearly the most sensitive of the CSF neuronal biomarkers and thus the most valuable for detecting HIV-related neurodegeneration over the course of infection. Not only were NFL concentrations abnormally high in all of the HAD patients, they revealed a substantial prevalence of less evident or subclinical injury in the NA groups with low blood CD4 T-cell counts as well as during the early phase of infection. In that sense, NFL serves as a standard for detecting and measuring active CNS injury in untreated HIV infection. Indeed, this was the basis of its use in regrouping the NA subjects with <200 CD4+ T cells into the NFL+ and NFL- groups. Of course, elevations of CSF NFL concentrations are not specific to HIV-related brain injury; CSF NFL concentrations have been shown to be a sensitive indictor of CNS axonal injury in a variety of neurological diseases [56], [58], [74]–[77] including various infections [46], [78]–[82], so other causes of ongoing injury need to be considered in individual cases with elevated CSF NFL. The subjects in this series were screened for opportunistic and other CNS diseases. The high prevalence of abnormal CSF NFL in the NA groups, increasing as CD4+ cell counts were reduced, along with a phase of early injury during PHI importantly documents the frequency of active neuronal injury in these patients and supports and supplements the findings of previous studies [4], [51], [60].

NFL is the light subunit of the neurofilament protein, a major structural component of myelinated axons that is essential for molecular transport over the length of the axon and for normal nerve conduction [83]. The sensitivity of NFL in detecting injury in HAD and non-HAD patients suggests that injury to myelinated axons is a common component of HIV-associated CNS damage that occurs early and continues in its most severe forms. This is also supported by neuropathological findings of axonal loss [84]. The correlation of elevated CSF NFL and reduced performance on the QNPZ-4 testing, including in some of the NA+ subjects, demonstrates that elevated NFL was associated with functional changes as systemic disease advanced, which in some patients was either overlooked on clinical evaluation or dismissed as related to other, usually static, conditions.

#### Tau proteins

CSF t-tau concentrations were frequently increased in the HAD group but not in those with low blood CD4+ T cell levels initially categorized as NA who had increased CSF NFL. P-tau was mainly present in normal concentrations in all of the subject groups, with low-level elevation in the HAD group that likely was secondary to the elevated t-tau and, in fact, was ‘disproportionately’ low in relation to t-tau compared to the other groups. These finding are compatible with previous reports, with differences related mainly to variations in diagnostic criteria, treatment status and interpretation [46], [47], [64]. Tau protein is expressed primarily in non-myelinated cortical axons. When the tau protein is hyperphosphorylated, it results in the detachment of tau from the microtubules, which destabilizes the axons and can lead to self-assembly of neurofibrillary tangles, a pathological hallmark of Alzheimer's diseases and other tauopathies [62], [63], [85]. This process is thus not a feature of HIV-related CNS damage.

Enhanced leakage of t-tau into CSF without a concomitant increase in p-tau in HAD likely signaled non-specific damage of cortical axons and neurons rather than a primary or selective effect on t-tau metabolism. This is similar to findings in other infections [48]. Elevation of t-tau only in HAD suggests that this type of injury is associated with CSF-related inflammation and blood-brain barrier damage, differing both from the inflammation of earlier infection (as in the NFL- and CD4 >200 groups) and the damage that occurs in the absence of pleocytosis (as in the NFL+ group). Further study of these distinctions is needed to understand the viral-immune-neuronal interactions distinguishing these different pathological processes.

#### Amyloid metabolites

CNS amyloid metabolism was also altered by HIV infection with significant reductions in CSF sAPPβ and to a lesser extent sAPPα concentrations in HAD without substantial parallel changes in Aβ peptides (Fig. 1, Table 3). Amyloid precursor protein (APP) is ubiquitously expressed in neurons and undergoes sequential proteolytic cleavage by different secretases resulting in metabolites that have been linked to a variety of neuropathological consequences [65]. The cleavage of APP by α-secretase and β-secretase results in production of soluble sAPPα and sAPPβ, respectively, which are both shed from the cell membrane and diffuse into the CSF. Intermediate reductions of these two APPs were also noted in the NFL+ group (Fig. 3) indicating onset of altered APP metabolism without overt clinical HAD. Reduction in these sAPPs was not noted in the PHI group despite elevated NFL, suggesting that there may have been differences in the pathophysiology of injury in these settings, though possibly this relates simply to differences in the magnitude of injury and requires further study [86].

These sAPP changes contrasted with the normal CSF levels of three different Aβ peptides (Aβ42 measured by the INNOTEST and Aβ42, Aβ40 and Aβ38 measured using the Triplex assay). Concentrations of these Aβ peptides were similar in all the studied groups, with Aβ42 actually increasing somewhat with CD4 progression across the NA and HAD groups unexpectedly correlating directly with CSF t-tau concentrations. While reported changes in CSF Aβ peptides have been varied [47], [64], [86], the results of this study are similar to findings reported in our earlier study that focused on HAD [46].

As previously hypothesized, the current findings suggest that CNS HIV infection alters APP processing or transport [46]. This is also consistent with pathological documentation of APP accumulation in axons in both HIV encephalitis [87] and a simian immunodeficiency virus (SIV) model of lentivirus encephalitis [88]. These changes in APP metabolism are not accompanied by accumulation of fibrillary Aβ fragments in the characteristic plaques that has been linked to neurodegeneration in Alzheimer's disease [66], and thus the pathogenesis of altered amyloid metabolism and brain injury in HIV infection is likely quite distinct from Alzheimer's disease.

### Application of neuronal biomarkers to clinical research and management

The CSF neuronal biomarkers applied to this study also may be useful in selected HIV clinical research and clinical management settings, including in the identification and classification of patients with active CNS injury and in differential diagnosis of patients presenting with neurological deterioration.

Since NFL is the most sensitive of the neuronal biomarkers studied in detecting the presence of active HIV-related CNS injury, it should be the most useful in assessing the presence and magnitude of active HIV-related injury. Settings where this might be clinically useful include untreated patients with background confounders that obscure whether neurological signs and symptoms are explained by active HIV-induced CNS disease. NFL may also reveal truly asymptomatic and therefore subclinical active CNS disease. In the research setting, this may be used to classify patients for studies that assess CNS efficacy of different treatment regimens or other issues related to neuronal injury.

In treated patients, elevated NFL may also be useful in characterizing and managing those with symptomatic CNS escapes [89], [90], though this needs further direct study. On the other hand, in the more common situation of treated-suppressed patients these biomarkers may not be sufficiently sensitive to detect mild ongoing injury in an individual patient, though group analysis may show differences from controls [52].

While CSF NFL is the most sensitive of these biomarkers, the others neuronal biomarkers may be useful for differential diagnosis, particularly for diagnosing Alzheimer's disease in an HIV-infected patient. This issue is likely to become more common as treatment extends the longevity of infected patients. Since HIV is not associated with notable elevation of CSF p-tau or depression in Aβ42 that characterize Alzheimer's disease, these biomarkers can be used to identify the latter in HIV patients suffering deteriorating cognition and help to monitor any increase in Alzheimer's disease prevalence in treated HIV infection.

### Study limitations and conclusions

There are a number of limitations to this study. While the overall number of subjects was relatively large, individual groups were limited in size, particularly the important HAD group and the minor elite group that were restricted by their low incidence and availability. Analysis included many comparisons of both the biomarkers and the groups, potentially resulting in both false positive and negative associations. For this reason some of the results should be considered more exploratory than definitive. They provide a broad overview of some essential aspects of disease progression and are helpful in formulating more targeted studies. The study used cross-sectional samples to reconstruct a longitudinal process. While longitudinal study would clearly be more desirable, logistically this will become increasingly difficult as guidelines call for earlier and broader treatment. The low proportion of women in the study across all the study groups also means that the results cannot yet be clearly applied to women with HIV infection. Despite these limitations, the study raises a number of interesting points, including:

- There was a seeming hierarchy of detection of neuronal injury with the sensitivity of CSF NFL > CSF sAPPs (particularly sAPPβ) > CSF t-tau as CD4 fell and HAD developed. This may reflect difference in injury mechanisms, patterns, or distributions, or alternatively in the catabolism and other properties of these biomarkers after release into the CSF compartment.
- NFL detected mild injury during PHI and in an increasing proportion of individuals as CD4 counts were reduced in the absence of overt HAD, and in those with overt HAD.
- The pattern of biomarker abnormalities in PHI with elevated CSF NFL but without depressed sAPPs may suggest a different mechanism of injury during this stage than in later infection.
- HAD was characterized not only by higher CSF NFL, but by t-tau elevation and both elevated CSF WBC counts and blood-brain barrier breakdown that distinguished it biologically from nonHAD neuronal injury detected in the low-CD4+ NFL+ group.
- Altered tau metabolism in HAD (high CSF t-tau with relatively reduced p-tau) likely reflects nonspecific injury of nonmyelinated proximal axons and neuronal cell bodies, differing from AD with the latter's associated neurofibrillary tangles.
- The profile of CSF biomarker changes in HIV-related CNS injury is quite different from that of Alzheimer's disease and can be used to distinguish these dementing conditions. There was no reduction in Aβ42 or the other measured Aβ fragments despite the reduction in sAPPβ, nor was there a distinct elevation of p-tau in any of the HIV-infected groups, including HAD, consistent with other evidence that HAD brain injury is very different from that of Alzheimer's disease.
- CSF sAPPs showed an intermediate pattern of reduction, less common and marked than NFL elevation with reduced blood CD4+ counts in the absence of HAD, but trending downward. Reduced CSF sAPPs may reflect a later more severe phase in brain injury than detected by NFL elevation but may also result in part from the difficulty of detecting an overall reduction compared to an increase in the release of a neuronal metabolite.
- Treatment-induced viral suppression was associated with normal or near normal CSF NFL. Since the individuals in this group experienced low CD4 nadirs, it is likely that some had suffered subclinical CSF NFL elevations in the past, and hence this had normalized with treatment (though a larger group analysis has suggested that CSF NFL may not fully reach age-adjusted control levels [52]).
- Classification of subjects combining blood CD4+ T cell counts and CSF NFL concentrations appears helpful in examining other factors associated with or contributing to CNS injury and thus may be useful in future studies.

## Methods

### Study Design

This was a cross-sectional study using archived CSF samples derived from two cohort studies: one in San Francisco, California and the second in Gothenburg, Sweden. The samples and related background data were all obtained between 1994 and 2010 within the context of research protocols approved by the institutional review boards of the two study sites and one of the funding sites; informed consent was obtained from all subjects. The studies were approved by: the University of California San Francisco Committee on Human Research (the UCSF IRB); the Regional Ethics Committée Gothenburg, the IRB used by the Sahlgrenska Hospital and University of Gothenburg; and University of California Davis Institutional Review Board (the IRB of UC Davis). All CSF and blood samples were performed with written consent of subjects under one these IRB-approved protocols.

CSF along with blood samples for assessment of background HIV status were included from 8 defined HIV-infected subject groups: early or ‘primary’ HIV infection (*PHI*, defined as within the first twelve months after initial HIV-1 infection) [2]; four groups of chronically HIV- infected subject volunteers without a diagnosis of HAD and who were not being evaluated or treated for overt neurological symptoms or signs when recruited, designated as *neuroasymptomatic* (*NA*) and divided by blood CD4+ T cell counts into those with >350, 200–349, 50–199, and CD4 <50 cells per µL; and a group presenting with clinically overt *HAD*. All of these subjects were either naïve to treatment or off treatment for at least 6 months at the time of sampling. Also included was a group of treated HIV+ subjects with ≥1 year of plasma virus suppression to below 50 copies/mL of HIV RNA (*Rx suppressed*) and a small group of untreated patients with documented HIV infection but plasma HIV RNA levels below 50 copies/mL (elite controllers, or *elites*). A group of uninfected (*HIV-*) volunteer subjects, confirmed by serological testing at study visit, were recruited from the San Francisco community for research assessments to provide comparison data for the HIV-infected subjects.

### CSF and Blood Sampling

CSF was obtained according to standard protocols as previously described [8], [91]. Subjects also underwent phlebotomy for concurrent blood sampling along with general medical and neurological assessments at the study visit as previously described [92]. CSF was placed immediately on wet ice and subsequently subjected to low-speed centrifugation to remove cells, aliquoted and stored within 2 hours of collection at ≤−70°C until the time of HIV RNA and the neuronal biomarker assays. Blood was collected in EDTA and similarly processed with plasma, aliquoted and stored in parallel with CSF for later batch assays.

### Background Laboratory Methods

HIV RNA levels were measured in cell-free CSF and plasma at each site using the ultrasensitive Amplicor HIV Monitor assay (versions 1.0 and 1.5; Roche Molecular Diagnostic Systems, Branchburg, NJ), Cobas TaqMan RealTime HIV-1 (version 1 or 2; Hoffmann-La Roche, Basel, Switzerland) or the Abbott RealTime HIV-1 assay (Abbot Laboratories, Abbot Park, IL, USA). All recorded viral loads that were below the lower limit of quantitation (40 copies per mL) were standardized to a defined ‘floor’ value of 39 copies per mL for descriptive purposes. Each study visit included assessments by local clinical laboratories using routine methods to measure CSF white blood cell (WBC) count, CSF and blood albumin, and blood CD4+ and CD8+ T lymphocyte counts by flow cytometry. Some of the background characteristics of these subjects have been reported previously [10], [49].

### Clinical Evaluations

All subjects underwent routine clinical bedside screening for symptoms or signs of CNS opportunistic infections or other conditions that might impact CSF biomarker concentrations. Diagnosis of HAD was based on clinicians' assessment at presentation which was characteristically subacute, and met American Academy of Neurology criteria [28]. Many of these subjects were studied before publication of the more formal Frascati criteria [13] and were diagnosed with AIDS dementia complex (ADC) stages 1–4 [27] but met the functional criteria for the Frascati diagnosis of HAD without the requisite extensive formal neuropsychological assessment. Subjects in San Francisco but not Gothenburg also underwent brief quantitative neurological performance testing to derive an aggregate normalized Z score on four tests (QNPZ-4 averaging the normalized scores on grooved pegboard, digit symbol, finger tapping, and timed gait tests) as previously described [4], [93].

### Neuronal Biomarker Measurements

Neuronal biomarkers were assessed by enzyme-linked immunosorbent assays (ELISAs). CSF neurofilament light chain protein concentrations were measured using a sensitive sandwich method (NF-light ELISA kit, UmanDiagnostics AB, Umeå, Sweden). T-tau, p-tau181 and Aβ42 were measured using the INNOTEST ELISAs (INNOGENETICS, Gent, Belgium). Concentrations of sAPPα and sAPPβ were determined using the MSD sAPPα/sAPPβ Multiplex Assay (MSD, Rockville, MD). To further explore Aβ fragments, we also measured three separate cleavage products (Aβ42, 40 and 38) using the Abeta Triplex assay by MSD; unlike the INNOTEST antibody combination that is directed against the full Aβ1-42 molecule recognizing amino acids 1 and 42 in the sandwich, these recognize the specific C-terminal portions in combination with an N-terminal antibody (6E10) that reacts with amino acids 4 to 9. All samples were analyzed in a single run in the Laboratory of Neurochemistry at the University of Gothenburg by board-certified laboratory technicians blind to clinical data using a single batch of reagents for each assay; intra-assay coefficients of variation were below 10% for all analyses. Previously described laboratory cutoffs for CSF NFL, Aβ1-42, t-tau, and p-tau were used for descriptive analysis [52], [94]. Based on age-related reference values, normal cutoff values in CSF for NFL were defined as <380 ng/L (<30 years), <560 ng/L (30–39 years), <890 ng/L (40–59 years), and <1850 ng/L (>59 years). Normal values for t-tau were <300 ng/L (18–44 years) and <400 ng/L (>44 years); values for p-tau were <60 ng/L (20–59 years) and <80 ng/L (>60 years); and values for the INNOTEST Aβ42 were >530 ng/L, regardless of age. Reference values have not been established for sAPPα, sAPPβ, or Aβ42, Aβ40, and Aβ38 by the Triplex assay. Some of the CSF NFL results and results of the PHI subjects have been previously reported in a different context [4], [10], [49], including within a larger data set characterizing CSF in relation to age [48]; here, in an expanded number of subject groups we emphasize the comparison of NFL with the other neuronal biomarkers and group differences. Because of the age differences among the subjects and between groups, for some comparisons CSF NFL results were corrected for age to 49 years old (mid-range of the main ‘normal’ age range of 40–59 years for the laboratory with the normal limit at <890 ng/L) using a regression formula based on a larger sample set of HIV-uninfected controls [52].

### Statistical Analysis

Comparison of biomarker concentrations between all and selected subject groups to address the *a priori* questions used non-parametric methods including Mann-Whitney to compare two groups and Kruskal-Wallis with Dunn's post hoc test to compare three or more groups. These methods were used to focus on characteristics of these pathobiologically important subject groups representing different phases or states of HIV-infected individuals. Biomarker associations were analyzed across the entire sample set using Spearman's rank correlation. Descriptive statistics generally used medians with intraquartile ranges and the exploratory statistical comparisons used nonparametric methods because of the skewed distributions and inclusion of values below the quantitative ranges of some of the variables. All statistics were performed with GraphPad Prism (version 6.0, GraphPad Software, San Diego, USA).

## Supporting Information

S1 Table Full set of the data included in the analysis.
(XLSX)
